# Case Report: A Pathological Odyssey in Primary Cutaneous Apocrine Carcinoma

**DOI:** 10.12688/f1000research.135154.3

**Published:** 2023-11-08

**Authors:** Dr. Suhit Naseri, Samarth Shukla, Dr. Sourya Acharya, Sunita Vagha

**Affiliations:** 1 Department of Pathology, Jawaharlal Nehru Medical College, Datta Megha Institute of Higher Education and Research, Sawangi, Wardha, India; 2 Department of Medicine, Jawaharlal Nehru Medical College, Datta Megha Institute of Higher Education and Research, Sawangi, Wardha, India

**Keywords:** Apocrine carcinoma, Axillary mass, rare presentation, apocrine neoplasms, solid lesion, lymph node excision, malignant, surgical excision.

## Abstract

Apocrine carcinoma is an extremely rare malignant cutaneous neoplasm that usually arises in areas with a high density of apocrine glands. Diagnosis can be challenging as tumours share histological and immunophenotypic characteristics with them. At first evaluation, the disease is often assumed to be benign. There have been approximately 100 reports of apocrine neoplasms in the literature.

A 48-year-old male presented with a right axillary mass which increased in size over a period of 2 years. The patient was reported to have had ayurvedic therapy, but his swelling remained unchanged. Axillary lymph nodes were palpable. USG axilla suggested a well-defined fungating solid isoechoic lesion. USG neck did not reveal any abnormality. The mass was surgically excised as a whole by removing the overlying skin with margins and lymph node excision. The patient was diagnosed with primary apocrine carcinoma after surgical excision. The differentials include adenocarcinoma of breast and prostate and apocrine adenoma. There are no established standards for the care of this form of carcinoma due to its rarity and the absence of clinical studies. A literature evaluation and further reporting will aid in developing diagnostic standards and the most efficient treatment options.

## Introduction

Apocrine carcinoma of the axilla is a seldom-seen form of breast cancer that originates in the axillary sweat glands. This disease has been reported to be an invasive ductal carcinoma subtype, the most prevalent form of breast cancer among women. From a surgical perspective, apocrine carcinoma of the axilla may present as a palpable mass or a non-palpable abnormality on imaging studies, such as mammography or ultrasound. Treatment typically involves surgical excision of the tumor with clear margins, which may be followed by radiation therapy and/or systemic chemotherapy depending on the stage and biology of the cancer. From a pathological standpoint, Large, pleomorphic cells with an abundance of eosinophilic cytoplasm and prominent nucleoli are seen in axillary apocrine carcinomas. Apocrine carcinoma of the axilla is uncommon, but it can still be a clinically relevant diagnosis that has to be treated promptly and effectively to provide the patient the best chance of survival.

## Case report

**Figure 1.**
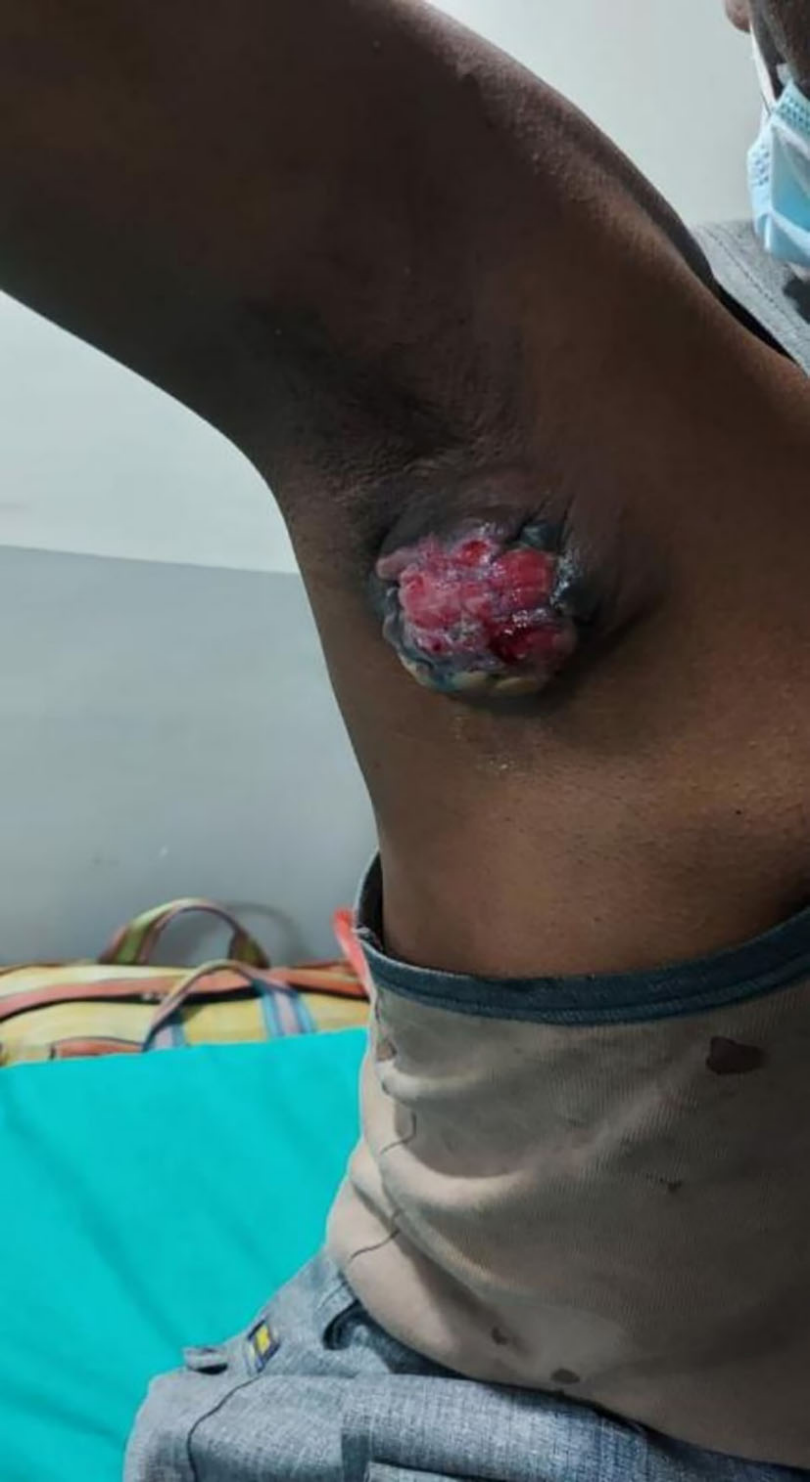
Clinical image of right axillary mass with inflammation.

In 2022, a male aged 48, hailing from South Asian origins and employed as a carpenter, visited the surgery department due to the presence of a lump ( Figure 1) in the axilla. The patient initially discovered a painless, indurated nodule in their right axilla around a year back. Over time, this nodule increased in size until the time of their presentation that is the size of 8 × 8 × 2.3 cm. The patient attempted ayurvedic treatment for a year, but the lesion continued to progress. On local examination, signs included inflammation of the skin covering the area, occasional discharge of a serosanguinous nature, and mild pain in the affected axilla. The patient did not experience any constitutional symptoms such as fever, weight loss, night sweats, or loss of appetite. The patient had no family history of malignancy. A physical evaluation and Ultrasonography (USG) for both breasts did not find any abnormalities. USG of the right axilla suggested a well-defined solid isoechoic lesion with multiple microcalcifications with prominent vascularity. Additionally, the patient had a preexisting condition of hypertension and was taking antihypertensive medications. Patient revealed his past habit of smoking bidi once a day. He did not consume alcohol.

A Tru Cut Biopsy was carried out which revealed cells showing moderate amount of eosinophillic to pale vaccuolated cytoplasm, Eccentric nuclei showing mild hyperchromasia and significant mitotic activity. Another population of cells seen with squamoid feature intervening the stroma showing desmoplastic reaction. Biopsy findings were suggestive of Malignancy of Adnexal origin.

Patient underwent wide local excision of the right axillary lesion with right axillary lymphadenectomy up to level III was carried out. A sufficient clearance margin of 1 - 1.5 cm was taken, and primary closure of defect was achieved.

Seven axillary lymph nodes were isolated, the largest lymph node was measured to be 4×2×1.5 cm.

**Figure 2.**
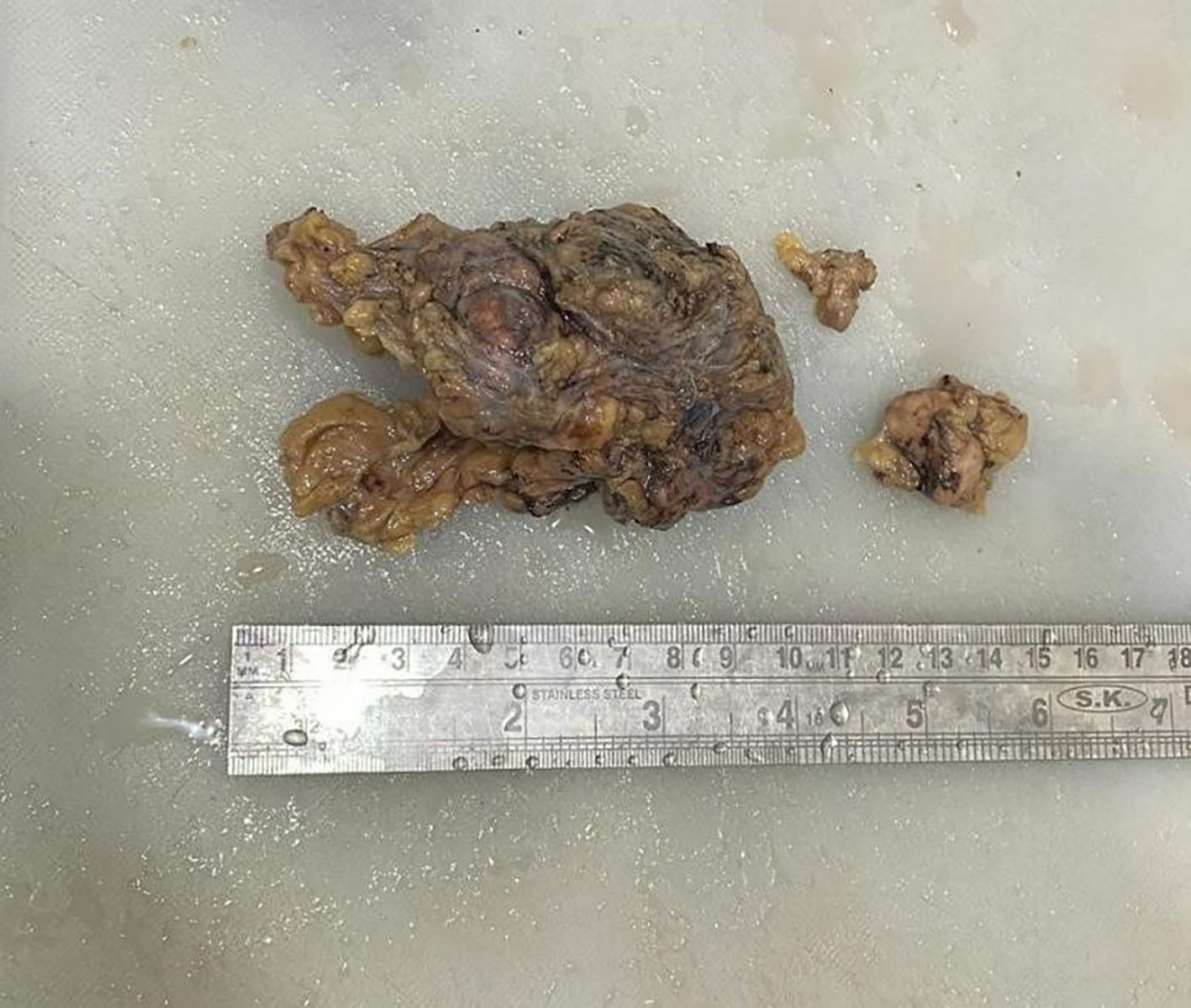
Gross image of excised specimen of axillary mass with a lymph node.

Grossly ( Figure 2), the tumour mass was white, firm in consistency and measured 4×4×3.5 cm. On the cut section, solid, homogenous blackish areas were identified with the involvement of overlying skin.

**Figure 3.**
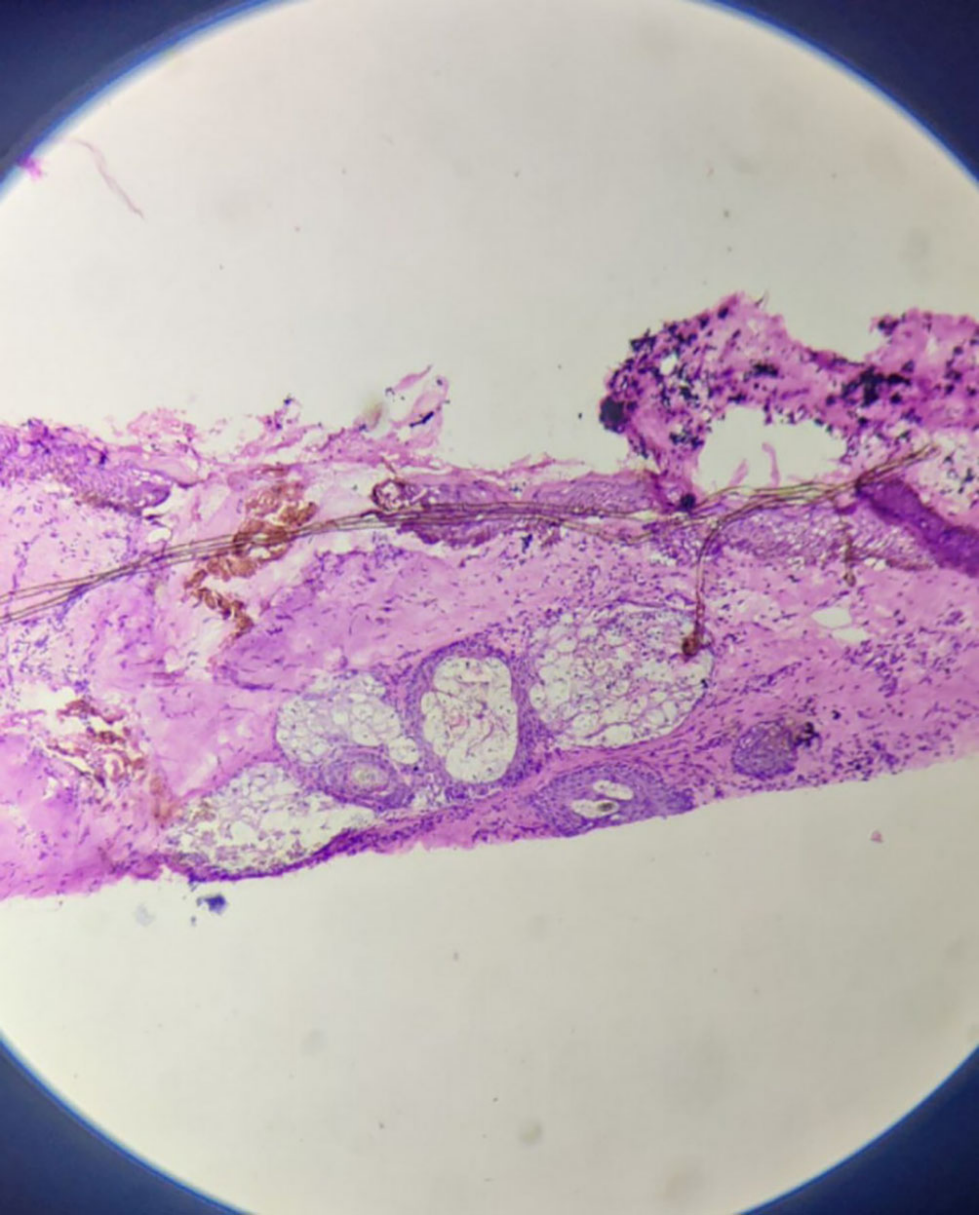
High power view of inferior surgical margin on frozen section – positive for infiltration by malignant cells.

Microscopically, sections from superior, anterior and posterior margins showed unremarkable squamous lining epithelium with unremarkable deeper tissue and adnexal structure with few distended ducts of histopathology. Section from the inferior margin ( Figure 3) was positive for infiltration by malignant epithelial cells. Sections also show fibro-collagenous areas with minimal scattered inflammatory infiltrate. Sections from the tumour were also positive for perineural and lymphovascular invasion.

Section from all seven lymph nodes shows histopathological features suggestive of metastatic deposits of epithelial malignancy.

The epidermis is visible in some portions of the tumour, which has a dermis with mostly papillary cystic architecture. Focal inflammation with numerous benign apocrine glands were noted.

**Figure 4.**
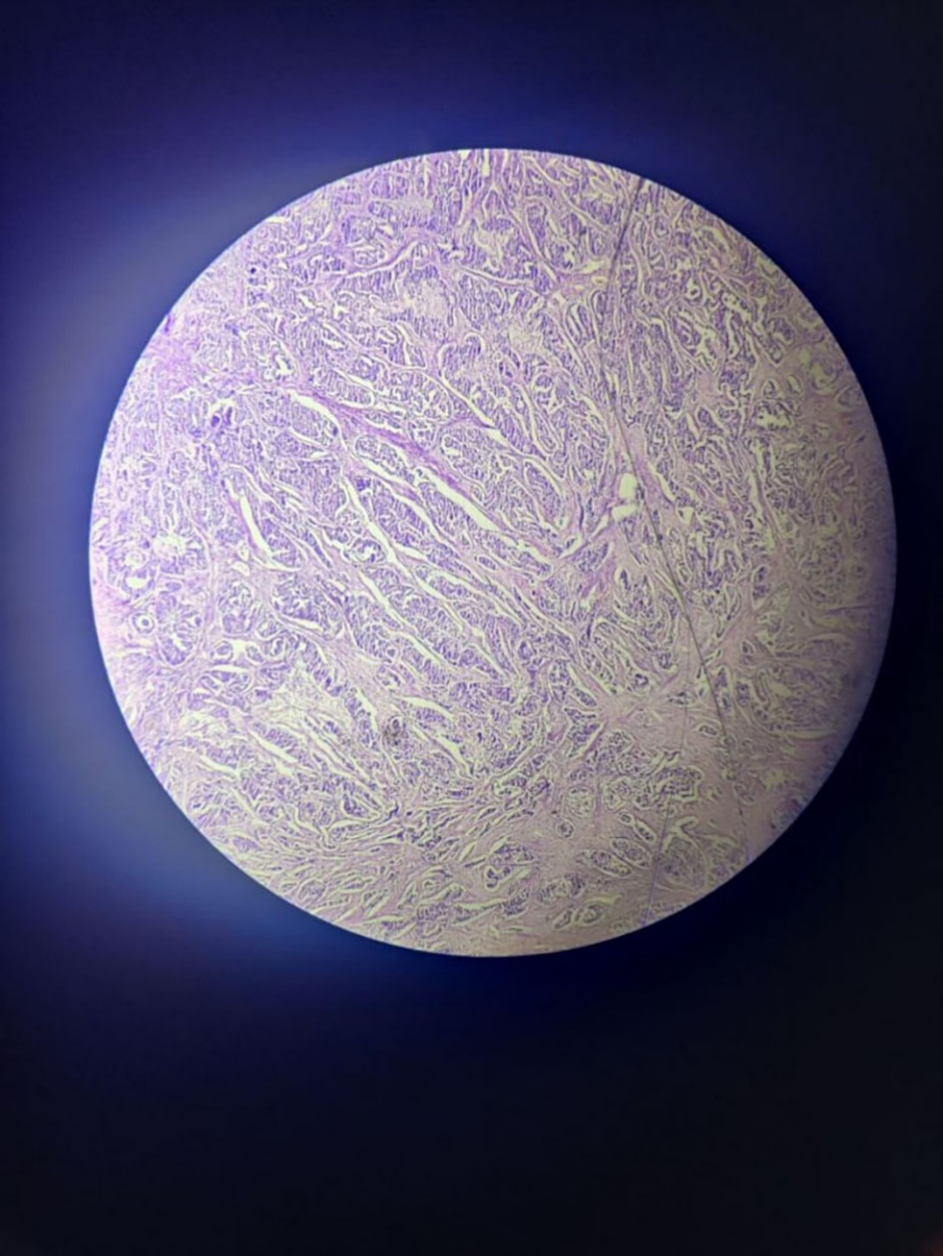
Tumor mass – scanner.

**Figure 5.**
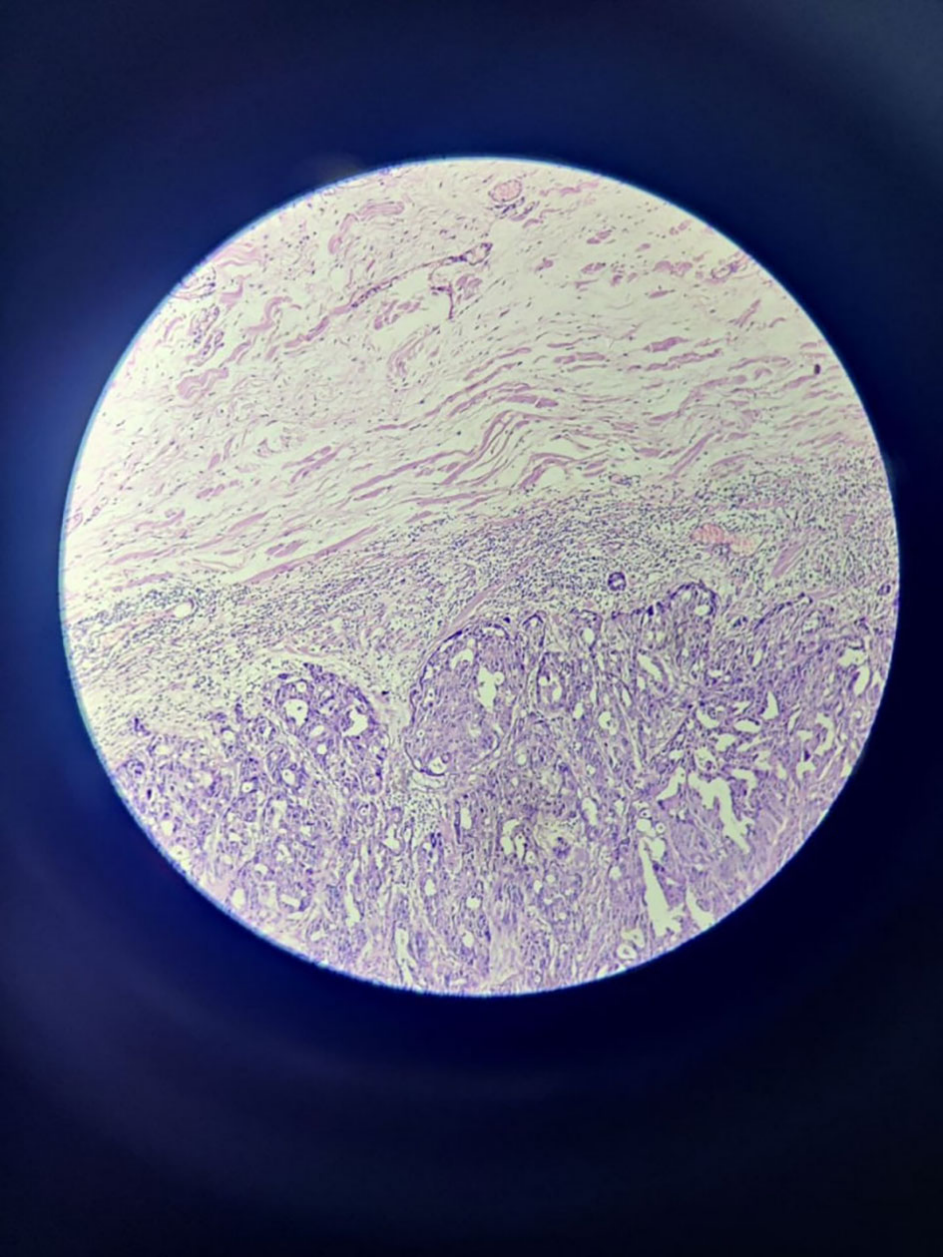
Tumor mass 10× – low power view.

**Figure 6.**
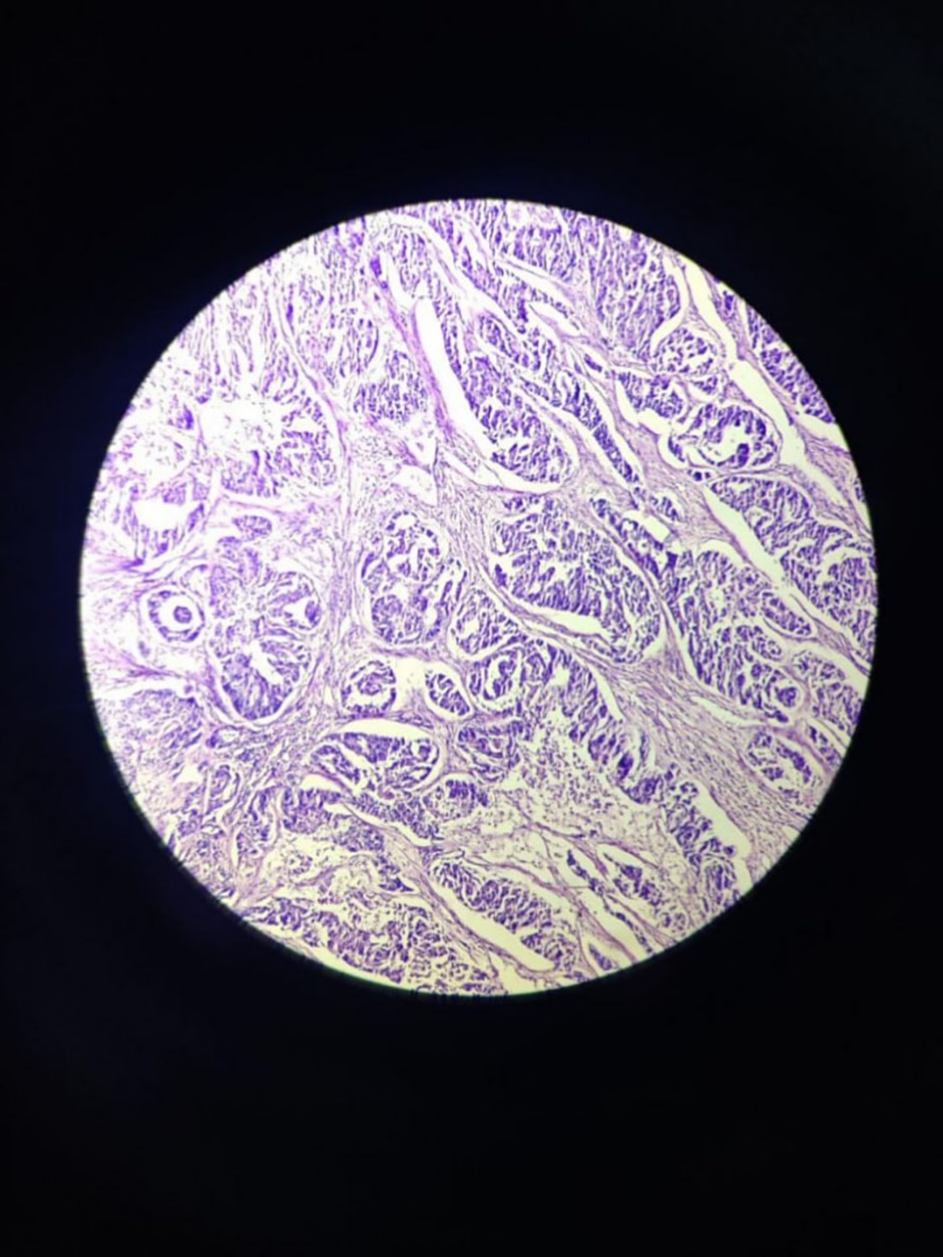
Tumor mass - 40× – high power view.

The tumour with papillary architecture ( Figures 4– 6) was found to have fibrovascular cores lined by eosinophilic epithelial cells. The patient's recuperation after surgery proceeded without any complications. The patient’s case was discussed in the hospital’s interdisciplinary tumour board and he was considered for adjuvant radiotherapy. Considering the metastatic involvement in the lymph nodes and to exclude the possibility of distant metastasis, a comprehensive whole-body Positron emission tomography–computed tomography (PET-CT) scan was performed, revealing no signs of distant metastatic spread.

A thorough follow-up plan was advised to the patient, but he did not follow through.

## Discussion

Apocrine carcinoma is an extremely uncommon adnexal malignancy with limited data on histologic prognostic factors and patient outcomes. ^1^ The axilla and adjacent medial upper arm are the most typical sites for apocrine carcinoma. ^2^ The tumour, which derives from pleuripotent adenexal cells capable of eccrine and follicular development, had first been discovered by Goldstein in 1982. ^3^ Reddish-purple subcutaneous nodules and solid or cystic masses are common characteristics of these tumours. Skin ulceration may be a comorbid condition, and they are frequently locally advanced when diagnosed. This tumour has a sluggish rate of growth, is locally invasive, and has the potential to spread to nearby lymph nodes, the lungs, the liver, the bone, and the brain. ^4^ When metastasis occurs, it occurs by lymphatic and hematogenous spread. ^4^ At the time of diagnosis, lymph node metastases were present in over fifty per cent of all reported individuals suffering from apocrine carcinoma, it is critical to consider the choice for SLN (sentinel lymph node) biopsy plus localised lymph node dissection. While there is still debate about the diagnostic criteria for apocrine carcinoma, decapitation secretion within eosinophilic epithelial cells is seen as an important confirmation of apocrine differentiation. If the SLN is positive, axillary lymphadenectomy ought to be performed. The recommended treatment for these lesions is wide local excision. ^5^ Apocrine gland carcinomas and eccrine carcinomas are the two primary subtypes of sweat gland carcinomas. Apocrine carcinomas appear as hard, rubbery, cystic, solitary or numerous, non-tender masses with red to purple overlaying skin. Eccrine gland carcinomas lack distinguishing clinical characteristics, rendering gross examination diagnosis nearly difficult. They often only affect older individuals and present as quasi-tender, subcutaneous nodules. ^6^ Although the tumour often arises de novo, it can potentially result from previously present benign tumours like apocrine hyperplasia or apocrine adenoma. ^7^ Several studies have shown that utilising CD15 and lysozyme staining can assist differentiate Apocrine malignancy from Eccrine malignancy. Furthermore, the presence of androgen receptors has been found to be closely linked to Apocrine carcinoma. In immunohistochemical analysis, several stains may be utilised to assist in determining the diagnosis of Apocrine carcinoma. 1,5 Cytokeratin AE1/AE3, CAM5.2, epithelial membrane antigen (EMA), smooth muscle antigen (SMA), periodic acid-Schiff positive with diastase resistance, and GCDFP-15 are all beneficial in confirming the diagnosis. ^8^ Wide local excision is the preferred method of management of primary cutaneous ductal apocrine carcinoma. In addition to excision, chemotherapy as well as radiotherapy have been utilised, but they haven’t significantly reduced mortality or morbidity among patients with either localised or metastatic disease. The total number of reported instances and the amount of follow-up data that currently exists, both seem insufficient for determining the prognosis. Therefore, there is a need for further case accumulation.

## Conclusion

The rare tumour referred to as apocrine carcinoma has a characteristic but non-specific histological appearance. For determining the prognosis and formulating particular therapy recommendations, the reported cases and follow-up data appear to be insufficient. Hence, additional cases needs to be collected.

## Consent

Written informed consent for publication of their clinical details and clinical images was obtained from the patient.

## Data Availability

All data underlying the results are available as part of the article and no additional source data are required.
