# Supplementary material for: Case Report: A Pathological Odyssey in Primary Cutaneous Apocrine Carcinoma
Source: F1000Res. 2023 Nov 8;12:803. Originally published 2023 Jul 10. [Version 3] doi: 10.12688/f1000research.135154.3 (PMC11176893; doi:10.12688/f1000research.135154.3)
Supplement: Peer review history

# Reviewer report: Reviewer response for version 3

**DOI:** 10.5256/f1000research.158522.r271478

**Published:** 2024-06-13

**Authors:** Dr. Ashwinkumar Barsagade

**Affiliations:** 1 Andaman and Nicobar Islands Institute of Medical sciences, Port Blair, India

This is a well written case report of rare malignancy, primary cutaneous apocrine carcinoma. the clinical findings, diagnostic workup including pathological findings and treatment are reported in detail. the case report in its discussion emphasizes the difficulties in recognizing and controlling this cancer due its rarity and inadequate prognostic data. The focus on the importance of incorporating apocrine carcinoma in the differential diagnosis of axillary tumors is helpful.

Are enough details provided of any physical examination and diagnostic tests, treatment given and outcomes?

Yes

Is the case presented with sufficient detail to be useful for other practitioners?

Yes

Is sufficient discussion included of the importance of the findings and their relevance to future understanding of disease processes, diagnosis or treatment?

Yes

Is the background of the case’s history and progression described in sufficient detail?

Yes

Reviewer Expertise:

Cytopathology and Histopathology

I confirm that I have read this submission and believe that I have an appropriate level of expertise to confirm that it is of an acceptable scientific standard.

# Reviewer report: Reviewer response for version 3

**DOI:** 10.5256/f1000research.158522.r221655

**Published:** 2023-11-30

**Authors:** Rehan Zahid

**Affiliations:** 1 Plastic Surgery, The University of Iowa Healthcare, Iowa City, IA, USA

I have reviewed the revisions. You can change the status to Approved. One thing I wasn't able to access was the list of references.

Are enough details provided of any physical examination and diagnostic tests, treatment given and outcomes?

No

Is the case presented with sufficient detail to be useful for other practitioners?

Partly

Is sufficient discussion included of the importance of the findings and their relevance to future understanding of disease processes, diagnosis or treatment?

Partly

Is the background of the case’s history and progression described in sufficient detail?

Partly

Reviewer Expertise:

NA

I confirm that I have read this submission and believe that I have an appropriate level of expertise to confirm that it is of an acceptable scientific standard.

# Reviewer report: Reviewer response for version 2

**DOI:** 10.5256/f1000research.156396.r202439

**Published:** 2023-10-03

**Authors:** Nour Kibbi

**Affiliations:** 1 Stanford University School of Medicine, Palo Alto, California, USA

This is a case report describing a rare presentation of a rare neoplasm. The authors should rename this as a primary cutaneous apocrine carcinoma since an invasive ductal breast carcinoma could not be found. The novelty of the case is not entirely clear and the authors might consider completing a literature review to determine what this case adds to the existing literature on this rare tumor. 

 Regarding language, the report is a bit wordy at times and would benefit from further editing for brevity and consistency. The histopathological images provided are not of adequate quality and would benefit from retaking with a camera attached to the microscope. There are abbreviations used without being spelled out, such as USG. It is also not clear what a Tru Cut biopsy is. The surgical margin is indicated as a range and it is not clear why.

Are enough details provided of any physical examination and diagnostic tests, treatment given and outcomes?

Yes

Is the case presented with sufficient detail to be useful for other practitioners?

Yes

Is sufficient discussion included of the importance of the findings and their relevance to future understanding of disease processes, diagnosis or treatment?

Yes

Is the background of the case’s history and progression described in sufficient detail?

Yes

Reviewer Expertise:

Rare tumors

I confirm that I have read this submission and believe that I have an appropriate level of expertise to confirm that it is of an acceptable scientific standard, however I have significant reservations, as outlined above.

# Response

**Published:** 2023-11-03

**Authors:** Dr. Suhit Naseri

**Affiliations:** Pathology, Jawaharlal Nehru Medical College, Datta Meghe Institue of Higher Education and Reaseach, Sawangi, Sawangi, Wardha, Maharashtra, India

*The authors should rename this as a primary cutaneous apocrine carcinoma since an invasive ductal breast carcinoma could not be found.*

 - Modification of the title is done.

*The novelty of the case is not entirely clear and the authors might consider completing a literature review to determine what this case adds to the existing literature on this rare tumor.*

 - Inclusion of further literature has been done. 

*Regarding language, the report is a bit wordy at times and would benefit from further editing for brevity and consistency.*

 - The required language corrections have been implemented.

*The histopathological images provided are not of adequate quality and would benefit from retaking with a camera attached to the microscope.*

 - Regrettably, due to the unavailability of the necessary resources, it is not feasible to retake the images.

*There are abbreviations used without being spelled out, such as USG. It is also not clear what a Tru Cut biopsy is. The surgical margin is indicated as a range and it is not clear why.*

 - Proper placement of abbreviations.

 - A Tru-Cut, also known as a Tru-Cut biopsy, is a medical procedure used to obtain a small core of tissue from a specific area of the body for diagnostic purposes.

 - The surgical margin is expressed as a range to account for variations in tumor size and shape.

# Reviewer report: Reviewer response for version 1

**DOI:** 10.5256/f1000research.148257.r192585

**Published:** 2023-08-24

**Authors:** Rehan Zahid

**Affiliations:** 1 Plastic Surgery, The University of Iowa Healthcare, Iowa City, IA, USA

I appreciate the authors for presenting this rare carcinoma. The authors have duly noted the lack of literature of such rare cases which makes it even more important to provide as much detail as possible when presenting this case.

 I suggest starting with elaborating the presentation. Progression of disease including a timeline and more details of the history and physical findings. Rephrase the first sentence of the case report section.

 Emphasize on the treatment plan. Was a biopsy done to confirm the diagnosis before the final excision? If it was planned as an excisional biopsy before being identified as a carcinoma then was a wide local excision carried out? Or planned? Especially with a positive inferior margin. Please clarify the surgical treatment that the patient underwent including lymph node dissection. 

 What were the surgical sequelae post excision? Was the defect closed primarily and was there any post surgical complication including wound healing delays or ulcerative margins? Although the patient was lost to follow up, it would be beneficial to provide as much data as available in terms of the outcome. It helps to identify the progression of disease and overall prognosis.  

 Was any other work up planned to check for metastatic disease? 

 Finally the authors should reflect on the findings of this case report and how it contributes to the existing literature on apocrine carcinoma. I feel there is more information that could be provided to contribute to the already scarce existing literature. Although it may not be practically feasible in many instances but all efforts should be made to pursue the patient for follow up and determine outcome of the disease.

Are enough details provided of any physical examination and diagnostic tests, treatment given and outcomes?

No

Is the case presented with sufficient detail to be useful for other practitioners?

Partly

Is sufficient discussion included of the importance of the findings and their relevance to future understanding of disease processes, diagnosis or treatment?

Partly

Is the background of the case’s history and progression described in sufficient detail?

Partly

Reviewer Expertise:

Plastic and Reconstructive Surgery

I confirm that I have read this submission and believe that I have an appropriate level of expertise to confirm that it is of an acceptable scientific standard, however I have significant reservations, as outlined above.

# Response

**Published:** 2023-09-21

**Authors:** Dr. Suhit Naseri

**Affiliations:** Pathology, Jawaharlal Nehru Medical College, Datta Meghe Institue of Higher Education and Reaseach, Sawangi, Sawangi, Wardha, Maharashtra, India

*1. Progression of disease including a timeline and more details of the history and physical findings.*

 In 2022, a male aged 48, hailing from South Asian origins and employed as a carpenter, visited the surgery department due to the presence of a lump (Figure 1) in the axilla. The patient initially discovered a painless, indurated nodule in their right axilla around a year back. Over time, this nodule increased in size until the time of their presentation that is the size of 8 x 8 x 2.3 cm. The patient attempted ayurvedic treatment for a year, but the lesion continued to progress. On local examination, signs included inflammation of the skin covering the area, occasional discharge of a serosanguinous nature, and mild pain in the affected axilla. The patient did not experience any constitutional symptoms such as fever, weight loss, night sweats, or loss of appetite. The patient had no family history of malignancy. A physical evaluation and USG for both breasts did not find any abnormalities. USG of the right axilla suggested a well-defined solid isoechoic lesion with multiple microcalcifications with prominent vascularity. Additionally, the patient had a preexisting condition of hypertension and was taking antihypertensive medications. Patient revealed his past habit of smoking bidi once a day. He did not consume alcohol.

*2. Emphasize on the treatment plan. Was a biopsy done to confirm the diagnosis before the final excision? If it was planned as an excisional biopsy before being identified as a carcinoma then was a wide local excision carried out? Or planned? Especially with a positive inferior margin. Please clarify the surgical treatment that the patient underwent including lymph node dissection.*

 A Tru Cut Biopsy was carried out which revealed cells showing moderate amount of eosinophillic to pale vaccuolated cytoplasm, Eccentric nuclei showing mild hyperchromasia and significant mitotic activity. Another population of cells seen with squamoid feature intervening the stroma showing desmoplastic reaction. Biopsy findings were suggestive of Malignancy of Adnexal origin.

 Patient underwent wide local excision of the right axillary lesion with right axillary lymphadenectomy up to level III was carried out.

*3. What were the surgical sequelae post excision?  The patient's recuperation after surgery proceeded without any complications.Was the defect closed primarily and was there any post surgical complication including wound healing delays or ulcerative margins?*

 A sufficient clearance margin of 1-1.5 cm was taken, and primary closure of defect was achieved. The patient's recuperation after surgery proceeded without any complications.

*4. Was any other work up planned to check for metastatic disease?*

 Considering the metastatic involvement in the lymph nodes and to exclude the possibility of distant metastasis, a comprehensive whole-body PET-CT scan was performed, revealing no signs of distant metastatic spread.
